# Maternal Preconception COVID-19 Vaccination and Its Protective Effect on Infants after a Breakthrough Infection during Pregnancy

**DOI:** 10.3390/vaccines12101132

**Published:** 2024-10-03

**Authors:** Yuting Yang, Jie Hu, Haijun Deng, Dapeng Chen, Guojin Wu, Huiwu Xing, Yuanyuan Liu, Shan Li, Yihan Yan, Ni Tang, Yao Zhao

**Affiliations:** 1 National Clinical Research Center for Child Health and Disorders, China International Science and Technology Cooperation Base of Child Development and Critical Disorders, Ministry of Education Key Laboratory of Child Development and Disorders, Chongqing Key Laboratory of Pediatric Metabolism and Inflammatory Diseases, Children’s Hospital of Chongqing Medical University, Chongqing 400015, China; yytcqmu@foxmail.com (Y.Y.); chendapeng@hospital.cqmu.edu.cn (D.C.); wuguojin0109@163.com (G.W.); xinghuiwu55@163.com (H.X.); 2 Key Laboratory of Molecular Biology for Infectious Diseases (Ministry of Education), Institute for Viral Hepatitis, Department of Infectious Diseases, The Second Affiliated Hospital, Chongqing Medical University, Chongqing 400016, China; hujie501@cqmu.edu.cn (J.H.); pepper027@163.com (H.D.); liuyuanyuan600599@163.com (Y.L.); andylee24@foxmail.com (S.L.); eliauk_han@163.com (Y.Y.); 3 Department of Laboratory Medicine, Bishan Hospital of Chongqing Medical University, Chongqing 402760, China

**Keywords:** SARS-CoV-2, vaccination, maternal immune, infants, protective effect

## Abstract

Background and aims: The transplacental vertical transfer of maternal antibodies was determined to be a crucial factor in conferring protective immunity to infants following delivery, and this study aimed to evaluate the protective effect of maternal preconception COVID-19 vaccination on infants. Methods: A prospective cohort study was conducted at the National Clinical Medical Research Center for Child Health and Diseases in Chongqing, China, spanning from July 2022 to April 2023. The study included infants from mothers with a preconception COVID-19 vaccination and (or) a SARS-CoV-2 infection during pregnancy. Titers of SARS-CoV-2 immunoglobulin G (IgG) and cross-neutralizing activity against SARS-CoV-2 variants were detected. Results: In this cohort study comprising 158 infants, it was observed that infants born to mothers who experienced a pregnancy-related breakthrough infection following a preconception vaccination had the highest titers of SARS-CoV-2 IgG and cross-neutralizing antibody activity against different variants compared to those with either of these factors alone. The transplacental vertical transmission of anti-SARS-CoV-2 antibodies decreased significantly with increasing age, from 3.16 ODs at birth to 2.29 ODs at two months, and persisted for approximately four months after birth. The predominant subclass of passively transmitted antibodies via the placenta was found to be IgG1, and a positive correlation was observed between the titers of SARS-CoV-2 IgG and IgG1 (R = 0.59, *p* < 0.001; Slope: 0.49 ± 0.070, *p* < 0.001). Conclusions: Maternal preconception COVID-19 vaccination represents a promising immunological strategy for conferring postnatal protection to infants, especially during the period of heightened risk of exposure to SARS-CoV-2 infection. It is imperative to underscore the significance of vaccination for women who are preparing to become pregnant or are pregnant, and concerted efforts must be made to promote vaccination among eligible women.

## 1. Introduction

Compared to older children, infants are at increased risk of experiencing hospitalizations related to Coronavirus Disease 2019 (COVID-19) complications, such as acute respiratory failure [1,2]. During the peak week of Omicron predominance, hospitalization rates for infants younger than 6 months of age were approximately six times higher than in the previous weeks [1]. To provide robust protection against severe illness stemming from SARS-CoV-2 infection, COVID-19 vaccines are the safest and most effective option available. Despite the authorization of messenger RNA (mRNA) COVID-19 vaccines for children as young as 6 months of age, the prevention of infection and illness in younger infants remains a significant concern.

Vaccinating women during pregnancy has been shown to effectively protect both the mother and her infant, as exemplified by combined tetanus, diphtheria, and pertussis (Tdap) vaccines, as well as influenza vaccines [3]. The transplacental transfer of immunoglobulin induced by an mRNA COVID-19 vaccination during pregnancy could be beneficial for conferring vertical immunity to fetuses and infants during early life [4,5,6]. Inactive COVID-19 vaccines are among the most commonly administered vaccine types, and are utilized in populations beyond pregnant women. In the previous study, we delineated the transfer of maternal anti-SARS-CoV-2 IgG, generated from COVID-19 vaccination before pregnancy, to infants through the placenta [7]. However, further investigation is required to ascertain the protective efficacy of maternal preconception vaccination on infants after birth.

According to the “National Report on the Epidemic of SARS-CoV-2 Infection” released by China Centers for Disease Control and Prevention (CDC), since December 2022, about 85% of people in China have been infected with SARS-CoV-2 [8]. China holds the top position globally in terms of the total number of COVID-19 vaccines administered [9]. Nevertheless, there is a dearth of data concerning the protective capacity of transplacental vertically transferred antibodies against SARS-CoV-2 variants in neonates born to mothers who received COVID-19 vaccination before pregnancy during the SARS-CoV-2 outbreak since December 2022 in China.

Gaining insight into the magnitude and breadth of maternal immunization through preconception vaccination is crucial for informing vaccination strategies for younger infants and bolstering pandemic readiness initiatives. This study aimed to investigate and compare the protective effect of anti-SARS-CoV-2 antibodies in infants born to mothers who received preconception vaccination and (or) experienced breakthrough infection during pregnancy, throughout the dominant phase of the SARS-CoV-2 Omicron variant.

## 2. Method

### 2.1. Study Design

A prospective study was designed and conducted at the National Clinical Medical Research Center for Child Health and Diseases in Chongqing, China, from July 2022 to April 2023. In order to elucidate the protective impact of the transplacental vertical transmission of anti-SARS-CoV-2 antibodies, we classified the infants based on the maternal preconception COVID-19 vaccination status and pregnancy-related breakthrough infection. A control group was established, consisting of infants from the pre-SARS-CoV-2 era and adult convalescent samples obtained from healthy volunteers. The study protocol was conducted in conformity with the Declaration of Helsinki for medical research involving human subjects and approved by the Ethical Committee of Children’s Hospital of Chongqing Medical University.

### 2.2. Sample Collection and SARS-CoV-2 Detection

Blood samples were procured from hospitalized infants at the National Clinical Medical Research Center for Child Health and Diseases, and subsequently centrifuged at 2500 rpm for 5 min at room temperature. Within two hours of completing the clinical program testing of the infant blood sample, a segment of the residual serum was aliquoted into cryogenic vials and stored at −80 °C for subsequent analysis. The demographic data of both mothers and infants were documented upon enrollment.

The diagnostic methods for SARS-CoV-2 infection were quantitative nucleic acid testing of nasopharyngeal swab sample in the laboratory and (or) rapid SARS-CoV-2 antigen tests (colloidal gold technique). Furthermore, to exclude SARS-CoV-2 infection in the infants, SARS-CoV-2 IgM was detected in all included samples.

### 2.3. SARS-CoV-2 RBD ELISA

Antibody level against the SARS-CoV-2 Receptor Binding Domain (RBD) protein in infants were quantified using an ELISA. Assays were performed in 96-well plates coated with 100 µL of recombinant RBD protein (Cat. #40592-V08B, Sino Biological, Beijing, China) diluted to 1 µg/mL in phosphate-buffered saline (PBS), and the plates were incubated at 4 °C overnight. The plates were washed with PBS-T (PBS containing 0.05% Tween-20) and then blocked for 2 h at room temperature (RT) with blocking buffer (PBS-T and 3% nonfat milk). Serum samples (1:3) and purified mAbs (Cat. #40150-D001, Sino Biological) were diluted in dilution buffer (PBST and 1% nonfat milk), and 100 µL of each sample was applied to a coated ELISA plate and incubated for 2 h at room temperature (RT). The plates were then washed with PBS-T. Secondary antibodies were diluted and incubated for 1 h at RT. For IgM, anti-human IgM (Cat. #ab97205, Abcam, Cambridge, UK) was diluted at a 1:10,000 dilution. For IgG, anti-human IgG (Cat. #109-035-088, Jackson ImmunoResearch, Pennsylvania, USA) was diluted at a 1:3000 dilution. For a subclass of IgG, anti-human IgG1, IgG2, IgG3, and IgG4 (Cat. #BF03069, #BF03066X, #BF03067X, #BF03068X, Biodragon, Suzhou, China) were diluted at a 1:3000 dilution. The plates were washed and developed with a tetramethylbenzidine (TMB) substrate and quenched with 1 M H_2_SO_4_. The samples’ optical density (OD) was measured by a spectrophotometer at 450 nm. The SARS-CoV-2 Spike antibody (Cat. #40150-D001, Sino Biological) was used as a positive control. The cut-off value was 0.05, calculated as 3 times the standard deviation of the OD values plus the mean of a large number of SARS-COV-2 negative serum. To ensure the accuracy of the test results, three replicate wells were set up for each sample and the average was taken as the reading for sample.

### 2.4. Production of SARS-CoV-2 S Pseudoviruses

In brief, to generate the SARS-CoV-2 S pseudoviruses with HIV-1 single-round luciferase, the pNL4-3.Luc.R-E-(The HIV-1 NL4-3 ΔEnv Vpr Luc reporter vector) and recombinant SARS-CoV-2 S plasmids (D614G, Alpha, Beta, Delta, Omicron BA.4/5, BF7, or XBB.1) were co-transfected into HEK293T cells using the Lipofectamine 8000 transfection reagent (beyotime, Shanghai, China). Fresh DMEM were added to the transfected cells 12 h later. The supernatant containing SARS-CoV-2 S pseudoviruses were harvested 60 h after transfection and filtered through a 0.45 μm filter [10].

### 2.5. Neutralization Assay

For the neutralization assay, 293T-ACE2 cells (1.5 × 104 cells/well) were seeded in 96-well plates; pseudoviruses were incubated with serial dilutions of sera samples (dilutions of 1:40, 160, 640, and 2560) from the infants grouped by the maternal vaccination and infection and convalescent adults for 1 h at 37 °C, then added to the 96-well 293T-ACE2 plates. Fresh culture medium was added to each well 12 h later. Luciferase activity was measured 60 h after infection and the neutralization titers was calculated using GraphPad Prism 8.0 software (GraphPad Software, San Diego, CA, USA). The titers of neutralizing antibodies were calculated as the 50% inhibitory dose (ID50), defined as the highest plasma dilution that resulted in a 50% reduction in luciferase luminescence compared to the virus control.

### 2.6. Statistical Analyses

The demographic characteristics of the infants and mothers were analyzed using descriptive statistics. Continuous variables were expressed as medians (interquartile range [IQR]), and categorical variables were expressed as numbers (%). Serum-neutralizing antibody levels were described as the geometric mean number with a 95% confidence interval. Neutralizing antibody titers were analyzed by plotting log2 values versus the fluorescein values (RLU) of plasma at different dilution ratios in GraphPad prism (version 9.0). The fold changes in antibody titers against various SARS-CoV-2 variants were determined for different groups. Differences between the two groups were assessed by the Mann–Whitney U test, and a 2-sided α of less than 0.05 was considered statistically significant. The correlation between the level of SARS-CoV-2 IgG antibodies and IgG1, time from infection to delivery, and IgG level were determined by two-tailed Pearson correlation test. Generalized Additive Model (GAM) was used to fit the non-linear curve between IgG and IgG1. The dynamic trends in IgG and serum-neutralizing antibody levels in relation to the age of the infants were analyzed by employing the Ordinary Least Squares (OLS) method and adjusting for sex, age of mother and trimester covariates in the R package rms. All statistical analyses were performed using R software, version 4.1.0 (R Foundation for Statistical Computing).

## 3. Results

**Figure 1:**
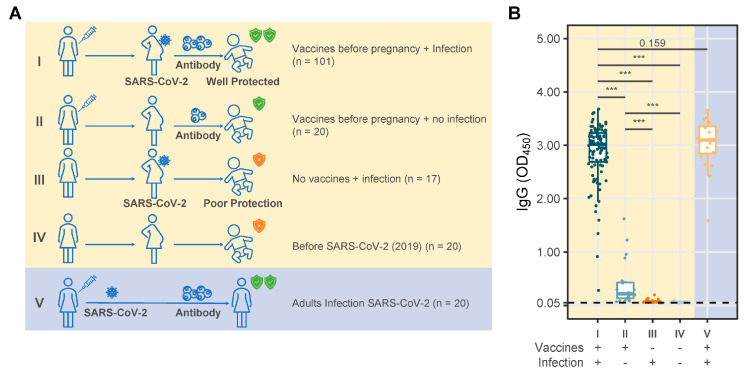
Study design and comparison of SARS-CoV-2 IgG titers. (**A**). The five cohorts used for immunological analysis are depicted. (**B**). Comparison the titers of SARS-CoV-2 IgG in infants stratifying by the maternal preconception COVID-19 vaccination status and infection during pregnancy, and with the control groups of infants from the pre-SARS-CoV-2 era and adult convalescents. Statistical significance labels: *p* ≥ 0.05 indicates no significant differences, *** indicates *p* < 0.001.

The research involved 178 of infants and 20 convalescent adults: (1) 101 infants were born to mothers who received COVID-19 vaccination before pregnancy and experienced a breakthrough infection during pregnancy; (2) 20 infants were born to mothers who were vaccinated before pregnancy and did not experience a breakthrough infection during pregnancy; (3) 17 infants were born to mothers who did not receive maternal COVID-19 vaccination before pregnancy and had SARS-CoV-2 infection during pregnancy; (4) 20 infants were from the pre-SARS-CoV-2 era; and (5) 20 serum samples were obtained from convalescent adults (Figure 1A, Table 1A). Following this, infants born to mothers who had received COVID-19 vaccination before pregnancy and experienced a breakthrough infection were categorized into three groups based on their age (<1 week, 1 month, and 2 months). The fundamental features of the subjects, including those in Table 1B, were outlined. None of the infants were found to be infected with SARS-CoV-2.

**Figure 2:**
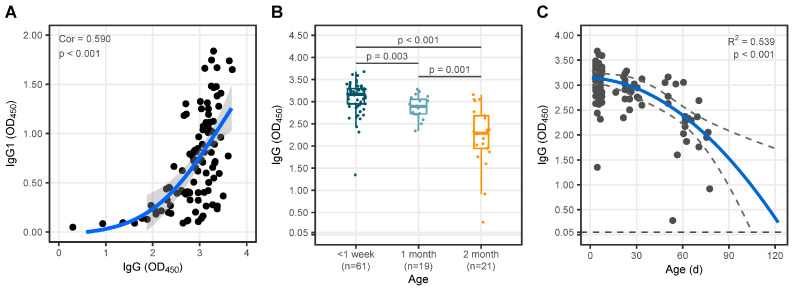
Analysis of the transplacental vertical transfer of IgG in infants with maternal preconception COVID-19 vaccination and breakthrough infection during pregnancy. (**A**) The positive association between IgG and IgG1 in infants, and the blue line is the fitted curve. (**B**) Comparison of IgG titers among groups of infants categorized by age: younger than 1 week, 1 month, and 2 months. (**C**). A fit curve (blue line) to depict the changes in SARS-CoV-2 IgG titers in infants with age.

A comparison of anti-SARS-CoV-2 IgG ODs among the groups of infants is presented in Figure 1B, and the corresponding descriptive statistical results are provided in Supplementary Table S1. Infants born to mothers who had received COVID-19 vaccination before pregnancy and experienced breakthrough infections during pregnancy exhibited the highest anti-SARS-CoV-2 immunoglobulin G (IgG) after birth. The IgG molecule comprises four distinct subclasses, which are differentiated by the structure of their constant regions. These subclasses include IgG1, IgG2, IgG3, and IgG4. The transplacental transmission of SARS-CoV-2 IgG subclasses from maternal to fetal circulation was predominantly composed of IgG1, with several instances of IgG2, IgG3, and (or) IgG4 positivity (as indicated in the Supplementary Table S2). The titers of SARS-CoV-2 IgG exhibited a positive correlation with the IgG1 subclass, R = 0.59, *p* < 0.001 (Figure 2A). We observed a significant decline in the transplacental vertical transfer of SARS-CoV-2 IgG levels in infants with increasing age, *p* < 0.05 (Figure 2B, Supplementary Table S3). The fitted curve demonstrated that the SARS-CoV-2 IgG levels in infants acquired through maternal transplacental transfer persisted for approximately four months postpartum (Figure 2C, R^2^ = 0.539, *p* < 0.001).

**Figure 3:**
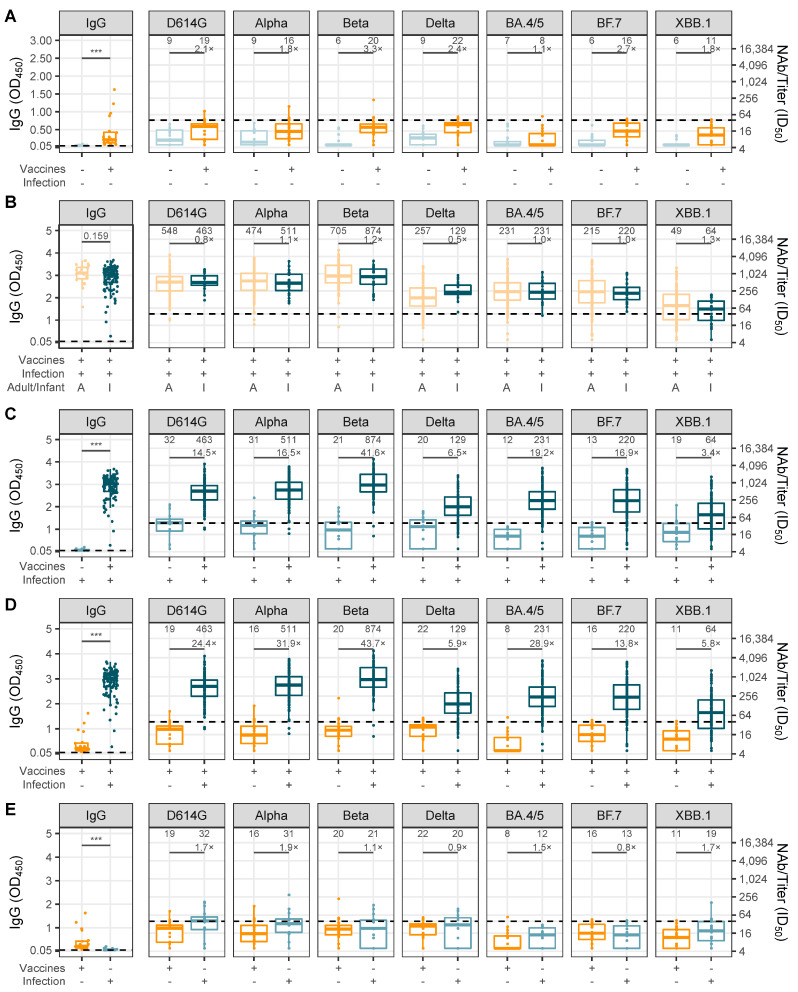
Pairing comparison of the titers of SARS-CoV-2 IgG and serum-neutralized antibody against D614G, Alpha, Beta, Delta, BA.4/5, BF.7, and XBB.1 variants in infants. (**A**) Comparison between infants born from the pre-SARS-CoV-2 era, and the mothers received COVID-19 vaccination before pregnancy and no breakthrough infection during pregnancy. (**B**) Comparison between the infants born to mothers received COVID-19 vaccination before pregnancy and infected during pregnancy, and the convalescent adults. (**C**) Comparison between infants born to mothers infected during pregnancy, with and without COVID-19 vaccination. (**D**) Comparison between infants born to mothers received vaccination before pregnancy with and without SARS-CoV-2 infection during pregnancy. (**E**) Comparison between infants born to mothers received vaccination before pregnancy and infected SARS-CoV-2 during pregnancy alone. The half-maximal inhibitory dose (ID50) was calculated as neutralizing antibody (NAb) titers. The threshold of ID50 detection was 1:40, which was represented by the dash line. Statistical significance labels: *** indicates *p* < 0.001.

To assess the protective efficacy of the transplacental transfer of anti-SARS-CoV-2 antibodies to infants, we conducted pairwise comparisons between groups with anti-SARS-CoV-2 IgG and cross-neutralizing antibodies against various variants (Supplementary Table S4). Infants born to mothers who received COVID-19 vaccination before pregnancy alone exhibited higher SARS-CoV-2 IgG levels and cross-neutralizing antibody titers against most SARS-CoV-2 variants compared to those born during the pre-SARS-CoV-2 period (Figure 3A). The titers of cross-neutralizing antibodies against the D614G, Alpha, and Beta variants in both infants and adult convalescents were found to be higher than those against the Delta, BA.4/5, BF.7, and XBB.1 variants of SARS-CoV-2 (Figure 3B). Infants born to mothers who had experienced post-vaccination breakthrough infections exhibited higher levels of SARS-CoV-2 IgG compared to infants whose mothers had not received maternal vaccination before pregnancy (*p* < 0.05). Furthermore, infants born to mothers who had experienced post-vaccination breakthrough infections had higher cross-neutralizing antibody titers than infants whose mothers had not received maternal vaccination before pregnancy. These titers increased by 18.1-, 22.9-, 54.7-, 9.6-, 29-, 23.5-, and 4.5-fold, respectively, for D614G, Alpha, Beta, Delta, BA.4/5, BF.7, and XBB.1 (Figure 3C). A comparable trend was observed between the cohort of infants born to mothers who received COVID-19 vaccination before pregnancy alone and those born to mothers who experienced post-vaccination breakthrough infection, with the latter group exhibiting a significant increase in cross-neutralizing antibody titers against various variants. Specifically, the fold increase in titers were 30.4-, 44.4-, 57.5-, 8.7-, 44.6-, 19.1-, and 7.8-fold, respectively (Figure 3D). Infants born to mothers who experienced SARS-CoV-2 breakthrough infections only during pregnancy exhibited higher cross-neutralizing antibody titers against most SARS-CoV-2 variants compared to infants whose mothers received vaccination only before pregnancy, increasing by 1.7-, 1.9-, 1.1-, 0.9-, 1.5-, 0.8-, 1.7-fold, respectively (Figure 3E). Additionally, infants born to mothers who received a three-dose COVID-19 vaccination regimen before pregnancy, particularly those who also experienced a breakthrough infection during pregnancy, demonstrated higher titers of SARS-CoV-2 IgG and cross-neutralizing antibodies against various variants compared to those born to mothers who received a 2-dose regimen (Supplementary Figure S1, Supplementary Table S5).

**Figure 4:**
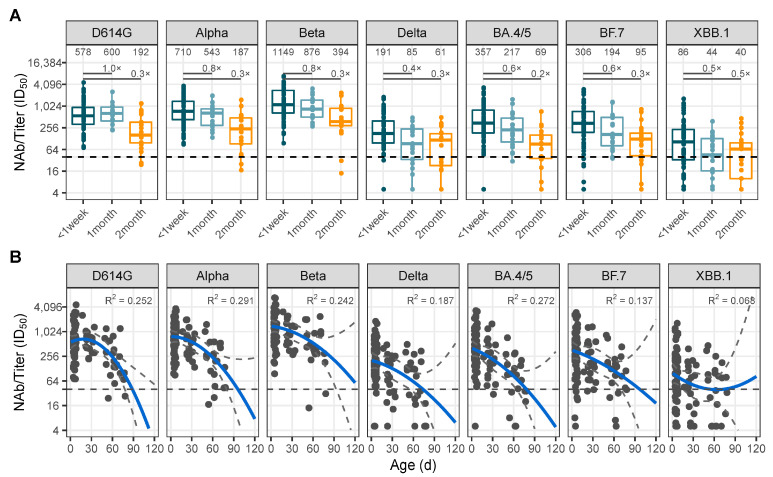
Analysis of the serum-neutralized antibody in infants with maternal preconception COVID-19 vaccination and breakthrough infection during pregnancy. (**A**) The changes in serum-neutralized antibody against D614G, Alpha, Beta, Delta, BA.4/5, BF.7, and XBB.1 variant in infants grouped by age: younger than 1 week, 1 month, and 2 months. (**B**) Fit curve of the changes in serum-neutralized antibody against D614G, Alpha, Beta, Delta, BA.4/5, BF.7, and XBB.1 variants in infants obtained by passive placental transfer decayed with age; the R^2^ was calculated. The threshold of ID50 detection was 1:40, which was represented by the dash line.

Transplacental vertical transfer of SARS-CoV-2 cross-neutralizing antibody titers in infants declined significantly with increasing age, and at 2-months of age, cross-neutralizing antibody titers against D614G, Alpha, Beta, Delta, BA.4/5, BF.7, and XBB.1 were decreased by 0.3-, 0.3-, 0.3-, 0.3-, 0.2-, 0.3-, and 0.5-fold, respectively (Figure 4A). The results of the fit curve indicated that cross-neutralizing activity against D614G, Alpha, Beta, Delta, BA.4/5, and BF.7 in infants acquiring through maternal transplacental transmission persisted around four months after birth (Figure 4B, Supplementary Table S6). The transplacental transfer of anti-SARS-CoV-2 antibodies through the placenta was not affected by the timing of maternal infection during pregnancy, and there was no significant disparity between the second and third trimesters (Supplementary Figure S2, Supplementary Table S7).

## 4. Discussion

This study determined that maternal breakthrough infection during pregnancy subsequent to preconception vaccination could potentially lower the likelihood of SARS-CoV-2 infection for infants who were at a heightened risk of infection from diverse SARS-CoV-2 variants. The levels of SARS-CoV-2 IgG antibody and cross-neutralizing activity in infants through transplacental vertical transmission exhibited a gradual and significant decrease after birth, but may still provide a substantial safeguarding effect during the initial four months of life.

Infants born to mothers who received COVID-19 vaccination before pregnancy and experienced breakthrough infections during pregnancy demonstrated significant cross-neutralizing efficacy and titers against various variants of concern. Prior research has indicated that the combination of COVID-19 vaccination and infection results in a heightened level of hybrid immunity, affording individuals a significant degree of protection [11,12,13,14,15]. Nevo et al. had reported that a booster dose of COVID-19 vaccine following SARS-CoV-2 infection could induce a robust increase in protective antibody titers for both mother and newborn [16]. Nevertheless, the extent to which transplacental transfer of anti-SARS-CoV-2 antibodies provides a safeguarding effect for infants born to women from a specific population who have experienced post-vaccination infection remains inadequately understood. In this study, we investigated the protective effect on infants using both ELISA and seven types of pseudotyped lentivirus neutralization assay. According to our study, infants born to mothers who received COVID-19 vaccination before experiencing a breakthrough infection during pregnancy exhibited a noteworthy elevation in anti-SARS-CoV-2 IgG and cross-neutralizing efficacy against diverse SARS-CoV-2 variants, possibly attributable to maternal pre-existing immune defenses. This discovery underscores the potential advantages of maternal preconception immunization in augmenting neonatal cross-neutralizing antibody response to COVID-19 variants.

Pertussis is a highly infectious respiratory disease, and maternal pertussis vaccination during pregnancy can provide infants with higher antibody levels at birth that persist for 2–3 months [3,17]. The protective impact of anti-SARS-CoV-2 antibody on infants acquired through maternal passive transmission diminishes gradually with age, and the reduction in the risk of a positive SARS-CoV-2 test may persist for up to the initial four months of life. Prior studies have investigated the durability of maternal protection against infections in infants resulting from maternal vaccination during pregnancy [18,19]. The results from our previous study showed that the passively acquired antibodies in infants may remain relatively stable regardless of the time interval between the mother’s last vaccine dose and pregnancy [6]. Notably, through the identification of the duration of cross-neutralizing antibody titers against various SARS-CoV-2 variants, the results of this study provide significant validation of the persistent protective impact observed in infants born to mothers who received preconception vaccination and encountered infection during pregnancy. The manifestation of a breakthrough SARS-CoV-2 infection among pregnant individuals who had undergone COVID-19 vaccination before pregnancy could trigger a strong maternal humoral immune response that endures throughout gestation, leading to a noteworthy enhancement of transplacental transmission of safeguarding anti-SARS-CoV-2 antibodies to the developing fetus. As a result, this highlights the far-reaching impact of maternal COVID-19 immunization on their progeny.

Interestingly, a breakthrough infection with the BA.5 or BF.7 Omicron variants can augment pre-existing immunity elicited by maternal vaccination before pregnancy. It is important to note that both the inactivated and recombinant COVID-19 vaccines employed are primarily formulated based on the ancestral isolate. In comparison to the spike protein of the Omicron variant, the spike proteins of the pre-Omicron variants exhibit greater similarity to the ancestral spike protein. Despite the predominance of the BA.5 and BF.7 strains during this epidemic since December 2022, COVID-19 vaccination has been shown to elicit pre-activated memory B cells that outcompete mutated variants-specific naïve B cells upon breakthrough infection with new mutated variants, thereby enhancing the production of high levels of antibodies against conserved epitopes [20]. The distinct spike mutations carried by various SARS-CoV-2 omicron sublineages may lead to the escape of XBB.1 from antibodies induced by breakthrough infections of BA.5 and BF.7 variants. Thus, the cross-neutralizing antibody titers of XBB.1 were found to be comparatively lower than those of the BA.5 or BF.7 variants. Furthermore, the efficacy of protection against the Delta variants was comparatively lower than that for other variants. This phenomenon may be attributed to the highly fusogenic nature of the Delta variant, as well as the presence of the P681R mutation in the spike protein, which can confer resistance to neutralizing antibodies [21].

Unvaccinated pregnant women are at a heightened risk of ICU admission, mechanical ventilation, and mortality from COVID-19 in comparison to appropriately matched non-pregnant women [22,23,24]. Rasmussen and Jamieson [25] have posited that COVID-19 during pregnancy is a “two for the price of one deal”. In the current investigation, we have demonstrated that the advantages of administering the COVID-19 vaccination before pregnancy are noteworthy for neonates postpartum. Furthermore, it has been observed that infants born to mothers with solely pregnancy-related SARS-CoV-2 infection exhibit reduced levels of anti-SARS-CoV-2 antibodies in comparison to those born to mothers who received vaccination before pregnancy. It has been posited that infection during pregnancy alone may elicit a restricted humoral immune response, which may not confer adequate protection to neonates against SARS-CoV-2 infection, irrespective of the viral variants

## 5. Strengths and Limitations

The present study has several strengths. This study provides insights into the protective effect of maternal preconception COVID-19 vaccination on neonates during the SARS-CoV-2 outbreak. We used well-established neutralization assays, complemented by serological assays, which allow for comparison with similar research and contribute to the robustness of our findings. However, there are several limitations to this study. First, a SARS-CoV-2 pseudovirus system was used to measure neutralizing antibody titers, rather than the live virus, which may not fully replicate the conditions of actual viral infection. Second, our study of antibody decay in infants was primarily based on cross-sectional age groupings, and we lacked long-term clinical follow-up of these infants. Additionally, the number of participants in our study was relatively limited, although we employed rigorous statistical methods in our data analysis and provided detailed explanations of the results. Finally, while the samples were collected from a single center, our study region encompassed a diverse population.

## 6. Conclusions

Our results enlarge our understanding of the substantial benefits of maternal COVID-19 vaccination before pregnancy, which could provide increased protection to infants following a maternal pregnancy-related breakthrough infection. Our findings highlight the importance of vaccination for women of childbearing age, and the necessity to increase efforts to encourage those who are preparing for pregnancy or gestating to be vaccinated, particularly as SARS-CoV-2 infections have become normalized.

## Figures and Tables

**Table 1:** (**A**) Clinical characteristics of individuals enrolled in this study. (**B**) Clinical characteristics of individuals from mothers who received COVID-19 vaccination before pregnancy and experienced breakthrough infection during pregnancy.

**(A)**			
**Characteristics**	**Vaccines before Pregnancy + Infection (Group I, *n* = 101)**	**Vaccines before Pregnancy + No Infection** **(Group II, *n* = 20)**	**No Vaccines + Infection (Group III, *n* = 17)**
Age (median, IQR)			
Mother (years)	30.0 (27.0–33.0)	29.0 (27.0–30.5)	33.0 (30.0–34.0)
Infant (days)	6.0 (4.0–30.0)	21.0 (3.8–41.0)	6.0 (3.0–7.0)
Gestational week (median, IQR)	39.0 (37.0–39.0)	39.0 (36.8–40.0)	38.0 (36.0–39.0)
Sex (Male/Female)			
Male	56 (55.4%)	10 (50.0%)	11 (64.7%)
Female	45 (44.6%)	10 (50.0%)	6 (35.3%)
Pregnancy trimesters			
Second	23 (22.8%)	-	6 (35.3%)
Third	78 (77.2%)	-	11 (64.7%)
Vaccination doses			
Unvaccinated (*n*, %)	0 (0.0%)	0 (0.0%)	17 (100.0%)
1 dose (*n*, %)	4 (4.0%)	0 (0.0%)	0 (0.0%)
2 dose (*n*, %)	31 (30.7%)	12 (60.0%)	0 (0.0%)
3 dose (*n*, %)	66 (65.3%)	8 (40.0%)	0 (0.0%)
IgG level (OD450)	3.02 (2.69–3.23)	0.22 (0.13–0.43)	0.07 (0.06–0.08)
**(B)**			
**Characteristics**	**Vaccines before Pregnancy + Infection (Group I, *n* = 101)**		
	**< 1 week (*n* = 61)**	**1 month (*n* = 19)**	**2 months (*n* = 21)**
Age (median, IQR)			
Mother (years)	30.0 (27.0–33.0)	30.0 (27.5–31.0)	30.0 (27.0–33.0)
Infant (days)	4.0 (3.0–6.0)	25.0 (22.0–29.0)	61.0 (55.0–69.0)
Gestational week (median, IQR)	39.0 (38.0–39.0)	38.5 (36.3–39.0)	38.0 (37.0–39.0)
Sex (Male/Female)			
Male	36 (59.0%)	8 (42.1%)	12 (57.1%)
Female	25 (41.0%)	11 (57.9%)	9 (42.9%)
Pregnancy trimesters			
Second	19 (31.1%)	1 (5.3%)	3 (14.3%)
Third	42 (68.9%)	18 (94.7%)	18 (85.7%)
Vaccination doses			
Unvaccinated (*n*, %)	0 (0.0%)	0 (0.0%)	0 (0.0%)
1 dose (*n*, %)	0 (0.0%)	2 (10.5%)	2 (9.5%)
2 dose (*n*, %)	18 (29.5%)	7 (36.8%)	6 (28.6%)
3 dose (*n*, %)	43 (70.5%)	10 (52.6%)	13 (61.9%)
IgG level (OD450)	3.16 (2.95–3.30)	2.90 (2.73–3.06)	2.29 (1.95–2.69)

## Data Availability

The data can be shared up on request.

## Supplementary Materials

The following supporting information can be downloaded at: https://www.mdpi.com/article/10.3390/vaccines12101132/s1, Table S1: The descriptive statistical results of anti-SARS-CoV-2 IgG in the participants; Table S2: The levels of anti-SARS-CoV-2 IgG subclasses in the participants; Table S3: The descriptive statistical results of anti-SARS-CoV-2 IgG levels in infants born to mothers who received COVID-19 vaccination before pregnancy and experienced breakthrough infection, grouped based on the infants’ age (<1 week, 1 month, and 2 months); Table S4: (a) The geometric mean titers (GMT) with 95% confidence interval (CI) of serum-neutralizing antibody in the participants; (b) The geometric mean ratio (GMR) with 95% CI of serum-neutralizing antibody in the participants; Table S5: (a) The geometric mean titers (GMT) with 95% CI of serum-neutralizing antibody in infants grouped by the maternal doses of COVID-19 vaccination before pregnancy; (b) The geometric mean ratio (GMR) with 95% CI of serum-neutralizing antibody in infants grouped by the maternal doses of COVID-19 vaccination before pregnancy; Table S6: (a) The geometric mean titers (GMT) with 95% CI of serum-neutralizing antibody in infants born to mothers who received COVID-19 vaccination before pregnancy and experienced breakthrough infection, grouped based on the infants’ age (<1 week, 1 month, and 2 months); (b) The geometric mean ratio (GMR) with 95% CI of serum-neutralizing antibody in infants born to mothers who received COVID-19 vaccination before pregnancy and experienced breakthrough infection, grouped based on the infants’ age (<1 week, 1 month, and 2 months); Table S7: (a) The geometric mean titers (GMT) with 95% CI of serum-neutralizing antibody in infants aged up to 1 week born to the mothers infected at the second (n = 19) or third trimester (n = 42); (b) The geometric mean ratio (GMR) with 95% CI of serum-neutralizing antibody in infants aged up to 1 week born to the mothers infected at the second (n = 19) or third trimester (n = 42); Figure S1: Comparison of SARS-CoV-2 IgG titers and serum-neutralized antibody against D614G, Alpha, Beta, Delta, BA.4/5, BF.7, and XBB.1 variants in infants grouped by the maternal doses of COVID-19 vaccination before pregnancy. (A) Comparison of SARS-CoV-2 IgG titers and serum-neutralized antibody against different variants in infants born to the mothers received 2- or 3-dose of vaccination before pregnancy and with no breakthrough infection during pregnancy. (B) Comparison of SARS-CoV-2 IgG titers and serum-neutralized antibody against different variants in infants born to the mothers received 2- or 3-dose of vaccination before pregnancy and with breakthrough infection during pregnancy; Figure S2: The titers of SARS-CoV-2 IgG and serum-neutralized antibody against D614G, Alpha, Beta, Delta, BA.4/5, BF.7, and XBB.1 variants in infants aged up to 1 week born to the mothers infected at the second (n = 19) or third trimester (n = 42). (A) The distribution of SARS-CoV-2 IgG titers in infants with the time difference from maternal infection to delivery; (B) Comparison of SARS-CoV-2 IgG titers in infants born to the mothers infected at the second or third trimester; (C) Comparison of serum-neutralized antibody against D614G, Alpha, Beta, Delta, BA.4/5, BF.7, and XBB.1 variants in infants born to the mothers infected at the second or third trimester
