# Supplementary material for: Maternal Preconception COVID-19 Vaccination and Its Protective Effect on Infants after a Breakthrough Infection during Pregnancy
Source: Vaccines (Basel). 2024 Oct 3;12(10):1132. doi: 10.3390/vaccines12101132 (PMC11511423; doi:10.3390/vaccines12101132)

Supplementary Table S1. The descriptive statistical results of anti-SARS-CoV-2 IgG in the participants.

IgG (OD_{450})	Vaccines before pregnancy + Infection (Group I, n=101)	Vaccines before pregnancy + no infection (Group II, n=20)	No vaccines + infection (Group III, n=17)	Before SARS-CoV-2 (2019) (Group IV, n=20)	Adults Infection SARS-CoV-2 (Group V, n=20)
Minimum value	0.281	0.070	0.048	0.046	1.588
25th percentile value	2.688	0.135	0.057	0.054	2.847
Median value	3.017	0.218	0.066	0.059	3.101
75th percentile value	3.230	0.429	0.084	0.065	3.355
Maximum value	3.678	1.623	0.198	0.100	3.655
Mean	2.866	0.400	0.081	0.061	3.044
SD	0.553	0.431	0.039	0.012	0.484

Supplementary Table S2. The levels of anti-SARS-CoV-2 IgG subclasses in the participants.

IgG subclass (OD450)	Vaccines before pregnancy + Infection (Group I, n=101)	Vaccines before pregnancy + no infection (Group II, n=20)	No vaccines + infection (Group III, n=17)	Before SARS-CoV-2 (2019) (Group IV, n=20)	Adults Infection SARS-CoV-2 (Group V, n=20)
IgG1	0.66 (0.32-1.07)	0.05 (0.05-0.06)	0.04 (0.04-0.05)	0.04 (0.04-0.05)	0.84 (0.6-1.22)
IgG2	0.06 (0.06-0.07)	0.04(0.04-0.05)	0.05 (0.05-0.07)	0.05 (0.05-0.07)	0.04(0.04-0.05)
IgG3	0.05 (0.05-0.06)	0.05 (0.04-0.06)	0.04 (0.04-0.05)	0.04 (0.04-0.05)	0.05 (0.04-0.06)
IgG4	0.05 (0.04-0.05)	0.05 (0.05-0.06)	0.05 (0.04-0.06)	0.05 (0.04-0.06)	0.05 (0.05-0.06)

Supplementary Table S3. The descriptive statistical results of anti-SARS-CoV-2 IgG levels in infants born to mothers who received COVID-19 vaccination before pregnancy and experienced breakthrough infection, grouped based on the infants' age (<≶1 week, 1 month, and 2 months).

IgG (OD_{450})	<1 week (n=61)	1-month (n=19)	2-month (n=21)
Minimum value	1.348	2.342	0.281
25th percentile value	2.950	2.726	1.948
Median value	3.164	2.895	2.293
75th percentile value	3.298	3.057	2.687
Maximum value	3.678	3.281	3.161
Mean	3.083	2.882	2.219
SD	0.361	0.249	0.707

Supplementary Table S4a. The geometric mean titers (GMT) with 95% confidence interval (CI) of serum-neutralizing antibody in the participants.

Strain	Nab/Titer (OD_{50})	Vaccinated & Infected mothers (Group I, n=101)	Uninfected mothers (Group II, n=20)	Unvaccinated mothers (Group III, n=17)	Pre-pandemic mothers (Group IV, n=20)	Adult convalescent (Group V, n=20)
**D614G**	**GMT**	**463**	**19**	**32**	**9**	**548**
	95% CI. Lower	376	13	18	7	403
	95% CI. Upper	569	27	58	13	746
**Alpha**	**GMT**	**511**	**16**	**31**	**9**	**474**
	95% CI. Lower	412	11	18	6	308
	95% CI. Upper	635	25	53	12	732
**Beta**	**GMT**	**874**	**20**	**21**	**6**	**705**
	95% CI. Lower	710	13	12	5	456
	95% CI. Upper	1077	31	37	8	1088
**Delta**	**GMT**	**129**	**22**	**20**	**9**	**257**
	95% CI. Lower	99	16	11	7	185
	95% CI. Upper	168	31	37	11	357
**BA.4/5**	**GMT**	**231**	**8**	**12**	**7**	**231**
	95% CI. Lower	177	6	8	5	154
	95% CI. Upper	300	12	17	10	346
**BF.7**	**GMT**	**220**	**16**	**13**	**6**	**215**
	95% CI. Lower	168	11	8	5	145
	95% CI. Upper	288	22	19	8	319

**XBB.1**	**GMT**	**64**	**11**	**19**	**6**	**49**
	95% CI. Lower	48	8	11	5	30
	95% CI. Upper	87	16	31	6	79

Supplementary Table S4b. The geometric mean ratio (GMR) with 95% CI of serum-neutralizing antibody in the participants.

Comparison	Values	D614G	Alpha	Beta	Delta	BA.4/5	BF.7	XBB.1
Group II vs. Group IV	GMR	2.11	1.78	3.33	2.44	1.14	2.67	1.83
	95% CI. Lower	1.41	1.19	2.22	1.63	0.76	1.78	1.22
	95% CI. Upper	3.16	2.66	5.00	3.66	1.71	4.00	2.75
Group I vs. Group V	GMR	0.84	1.08	1.24	0.50	1.00	1.02	1.31
	95% CI. Lower	0.56	0.72	0.83	0.33	0.67	0.68	0.87
	95% CI. Upper	1.27	1.62	1.86	0.75	1.50	1.53	1.96
Group I vs. Group III	GMR	14.47	16.48	41.62	6.45	19.25	16.92	3.37
	95% CI. Lower	9.65	11.00	27.77	4.30	12.84	11.29	2.25
	95% CI. Upper	21.69	24.71	62.39	9.67	28.86	25.37	5.05
Group I vs. Group II	GMR	24.37	31.94	43.70	5.86	28.88	13.75	5.82
	95% CI. Lower	16.26	21.31	29.15	3.91	19.26	9.17	3.88
	95% CI. Upper	36.53	47.87	65.51	8.79	43.28	20.61	8.72
Group III vs. Group II	GMR	1.68	1.94	1.05	0.91	1.50	0.81	1.73
	95% CI. Lower	1.12	1.29	0.70	0.61	1.00	0.54	1.15
	95% CI. Upper	2.52	2.90	1.57	1.36	2.25	1.22	2.59

Supplementary Table S5a. The geometric mean titers (GMT) with 95% CI of serum-neutralizing antibody in infants grouped by the maternal doses of COVID-19 vaccination before pregnancy.

Strain	Nab/Titer (OD_{50})	Group II		Group I	
		2-dose (n=12)	3-dose (n=8)	2-dose (n=31)	3-dose (n=66)
D614G	**GMT**	**14**	**28**	**307**	**573**
	95% CI. Lower	9	14	223	444
	95% CI. Upper	22	55	423	738
Alpha	**GMT**	**14**	**22**	**331**	**649**
	95% CI. Lower	8	10	227	506
	95% CI. Upper	24	47	482	832
Beta	**GMT**	**13**	**38**	**623**	**1054**
	95% CI. Lower	8	19	470	819
	95% CI. Upper	21	74	826	1358
Delta	**GMT**	**18**	**31**	**70**	**171**
	95% CI. Lower	11	22	42	126
	95% CI. Upper	29	44	115	232
BA.4/5	**GMT**	**9**	**8**	**136**	**297**
	95% CI. Lower	5	5	86	218
	95% CI. Upper	14	13	216	403
BF.7	**GMT**	**15**	**17**	**109**	**297**
	95% CI. Lower	9	8	67	218
	95% CI. Upper	24	34	176	404
XBB.1	**GMT**	**12**	**10**	**30**	**88**
	95% CI. Lower	7	6	18	62
	95% CI. Upper	20	17	51	126

Supplementary Table S5b. The geometric mean ratio (GMR) with 95% CI of serum-neutralizing antibody in infants grouped by the maternal doses of COVID-19 vaccination before pregnancy.

Comparison	Values	D614G	Alpha	Beta	Delta	BA.4/5	BF.7	XBB.1
3-dose vs. 2-dose in Group II	GMR	2.00	1.57	2.92	1.72	0.89	1.13	0.83
	95% CI. Lower	1.33	1.05	1.95	1.15	0.59	0.76	0.56
	95% CI. Upper	3.00	2.36	4.38	2.58	1.33	1.70	1.25
3-dose vs. 2-dose in Group I	GMR	1.87	1.96	1.69	2.44	2.18	2.72	2.93
	95% CI. Lower	1.25	1.31	1.13	1.63	1.46	1.82	1.96
	95% CI. Upper	2.80	2.94	2.54	3.66	3.27	4.08	4.40

Supplementary Table S6a. The geometric mean titers (GMT) with 95% CI of serum-neutralizing antibody in infants born to mothers who received COVID-19 vaccination before pregnancy and experienced breakthrough infection, grouped based on the infants' age (<1 week, 1 month, and 2 months).

Strain	Nab/Titer (OD_{50})	<1 week (n=61)	1 month (n=19)	2 month (n=21)
D614G	**GMT**	**578**	**600**	**192**
	95% CI. Lower	451	442	115
	95% CI. Upper	742	814	320
Alpha	**GMT**	**710**	**543**	**187**
	95% CI. Lower	559	375	107
	95% CI. Upper	901	786	326
Beta	**GMT**	**1149**	**876**	**394**
	95% CI. Lower	904	606	225
	95% CI. Upper	1461	1268	691
Delta	**GMT**	**191**	**85**	**61**
	95% CI. Lower	142	46	32
	95% CI. Upper	257	157	118
BA.4/5	**GMT**	**357**	**217**	**69**
	95% CI. Lower	266	129	37
	95% CI. Upper	477	368	127
BF.7	**GMT**	**306**	**194**	**95**
	95% CI. Lower	217	114	52
	95% CI. Upper	431	331	172

XBB.1	**GMT**	**86**	**44**	**40**
	95% CI. Lower	58	23	20
	95% CI. Upper	127	83	79

Supplementary Table S6b. The geometric mean ratio (GMR) with 95% CI of serum-neutralizing antibody in infants born to mothers who received COVID-19 vaccination before pregnancy and experienced breakthrough infection, grouped based on the infants' age (<1 week, 1 month, and 2 months).

Comparison	Values	D614G	Alpha	Beta	Delta	BA.4/5	BF.7	XBB.1
1month vs. <1week	GMR	1.04	0.76	0.76	0.45	0.61	0.63	0.51
	95% CI. Lower	0.69	0.51	0.51	0.30	0.41	0.42	0.34
	95% CI. Upper	1.56	1.15	1.14	0.67	0.91	0.95	0.77
2month vs. <1week	GMR	0.33	0.26	0.34	0.32	0.19	0.31	0.47
	95% CI. Lower	0.22	0.18	0.23	0.21	0.13	0.21	0.31
	95% CI. Upper	0.50	0.39	0.51	0.48	0.29	0.47	0.70

Supplementary Table S7a. The geometric mean titers (GMT) with 95% CI of serum-neutralizing antibody in infants aged up to 1 week born to the mothers infected at the second (n=19) or third trimester (n=42).

Strain	Nab/Titer (OD_{50})	Trimester with age<1week in Group I	
		Second trimester (n=19)	Third trimester (n=42)
D614G	**GMT**	**615**	**562**
	95% CI. Lower	365	420
	95% CI. Upper	1037	751
Alpha	**GMT**	**790**	**676**
	95% CI. Lower	491	509
	95% CI. Upper	1273	898
Beta	**GMT**	**1457**	**1032**
	95% CI. Lower	839	799
	95% CI. Upper	2532	1332
Delta	**GMT**	**226**	**177**
	95% CI. Lower	122	125
	95% CI. Upper	418	250
BA.4/5	**GMT**	**354**	**358**
	95% CI. Lower	172	265
	95% CI. Upper	726	483
BF.7	**GMT**	**337**	**292**
	95% CI. Lower	152	202
	95% CI. Upper	748	424
XBB.1	**GMT**	**103**	**79**

	95% CI. Lower	45	51
	95% CI. Upper	238	123

Supplementary Table S7b. The geometric mean ratio (GMR) with 95% CI of serum-neutralizing antibody in infants aged up to 1 week born to the mothers infected at the second (n=19) or third trimester (n=42).

Comparison	Values	D614G	Alpha	Beta	Delta	BA.4/5	BF.7	XBB.1
Third vs. Second Trimester with age<1week in Group I	GMR	0.91	0.86	0.71	0.78	1.01	0.87	0.77
	95% CI. Lower	0.61	0.57	0.47	0.52	0.67	0.58	0.51
	95% CI. Upper	1.37	1.28	1.06	1.17	1.52	1.30	1.15

Supplementary Figure S1. Comparison of SARS-CoV-2 IgG titers and serum-neutralized antibody against D614G, Alpha, Beta, Delta, BA.4/5, BF.7, and XBB.1 variants in infants grouped by the maternal doses of COVID-19 vaccination before pregnancy.

A). Comparison of SARS-CoV-2 IgG titers and serum-neutralized antibody against different variants in infants born to the mothers received 2- or 3-dose of vaccination before pregnancy and with no breakthrough infection during pregnancy.

B). Comparison of SARS-CoV-2 IgG titers and serum-neutralized antibody against different variants in infants born to the mothers received 2- or 3-dose of vaccination before pregnancy and with breakthrough infection during pregnancy.

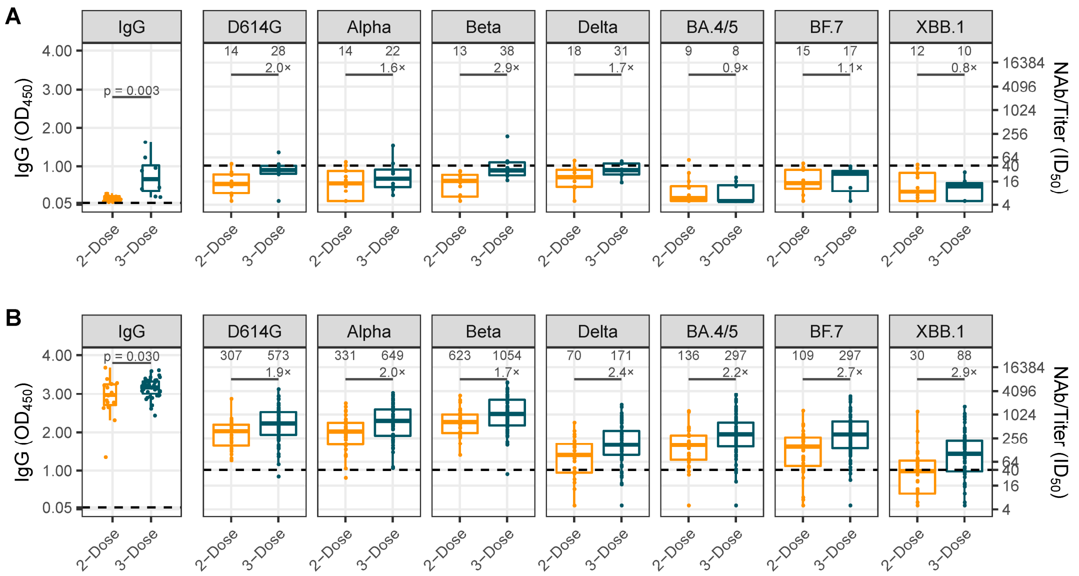

Supplementary Figure S2. The titers of SARS-CoV-2 IgG and serum-neutralized antibody against D614G, Alpha, Beta, Delta, BA.4/5, BF.7, and XBB.1 variants in infants aged up to 1 week born to the mothers infected at the second (n=19) or third trimester (n=42).

A). The distribution of SARS-CoV-2 IgG titers in infants with the time difference from maternal infection to delivery.

B). Comparison of SARS-CoV-2 IgG titers in infants born to the mothers infected at the second or third trimester.

C). Comparison of serum-neutralized antibody against D614G, Alpha, Beta, Delta, BA.4/5, BF.7, and XBB.1 variants in infants born to the mothers infected at the second or third trimester.

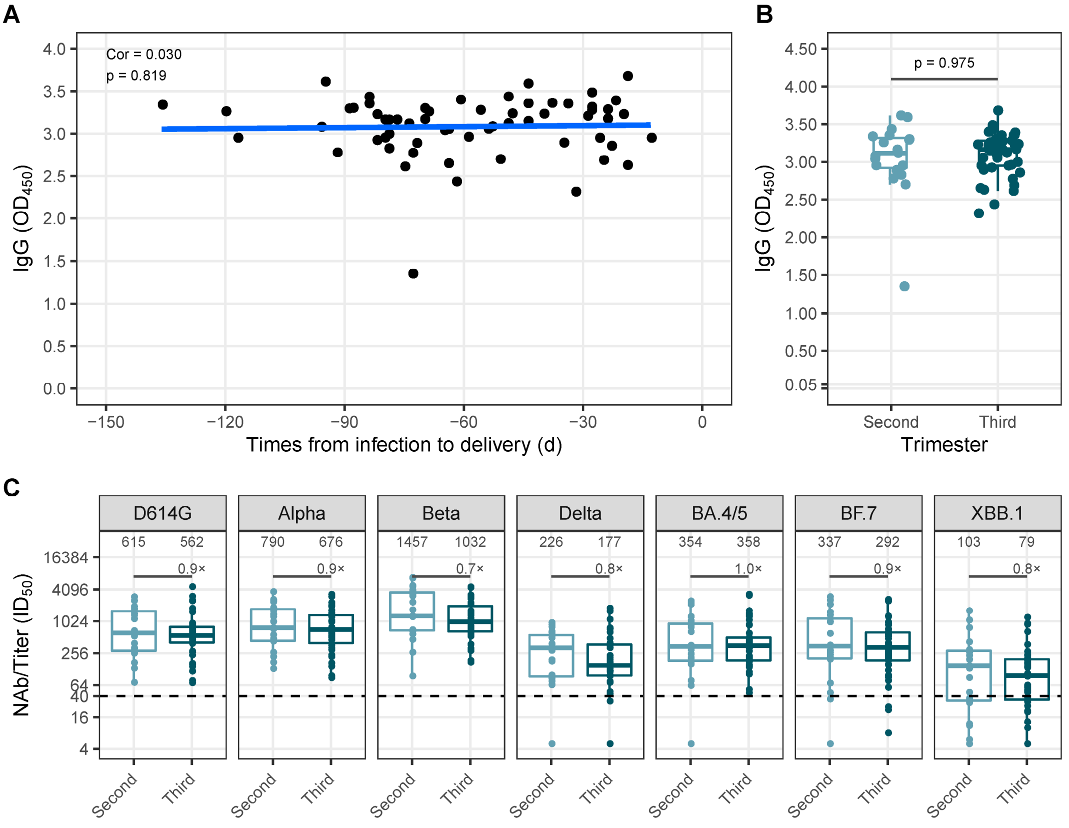

Supplement: Supplementary file 1 [file vaccines-12-01132-s001.zip › vaccines-3213160-supplementary.pdf]
